# Copula geoadditive modelling of anaemia and malaria in young children in Kenya, Malawi, Tanzania and Uganda

**DOI:** 10.1186/s41043-020-00217-8

**Published:** 2020-11-06

**Authors:** Danielle J. Roberts, Temesgen Zewotir

**Affiliations:** School of Mathematics, Statistics and Computer Science, University of KwaZulu-Natal, Durban, South Africa

**Keywords:** Joint modelling, Joint probabilities, Kendall’s tau, Spline smoothing

## Abstract

**Background:**

Anaemia and malaria are the leading causes of sub-Saharan African childhood morbidity and mortality. This study aimed to explore the complex relationship between anaemia and malaria in young children across the districts or counties of four contiguous sub-Saharan African countries, namely Kenya, Malawi, Tanzania and Uganda, while accounting for the effects of socio-economic, demographic and environmental factors. Geospatial maps were constructed to visualise the relationship between the two responses across the districts of the countries.

**Methods:**

A joint bivariate copula regression model was used, which estimates the correlation between the two responses conditional on the linear, non-linear and spatial effects of the explanatory variables considered. The copula framework allows the dependency structure between the responses to be isolated from their marginal distributions. The association between the two responses was set to vary according to the district of residence across the four countries.

**Results:**

The study revealed a positive association between anaemia and malaria throughout the districts, the strength of which varied across the districts of the four countries. Due to this heterogeneous association between anaemia and malaria, we further considered the joint probability of each combination of outcome of anaemia and malaria to further reveal more about the relationship between the responses. A considerable number of districts had a high joint probability of a child being anaemic but not having malaria. This might suggest the existence of other significant drivers of childhood anaemia in these districts.

**Conclusions:**

This study presents an alternative technique to joint modelling of anaemia and malaria in young children which assists in understanding more about their relationship compared to techniques of multivariate modelling. The approach used in this study can aid in visualising the relationship through mapping of their correlation and joint probabilities. These maps produced can then help policy makers target the correct set of interventions, or prevent the use of incorrect interventions, particularly for childhood anaemia, the causes of which are multiple and complex.

## Background

Anaemia and malaria are major contributors of childhood morbidity and mortality, particularly in sub-Saharan Africa [1, 2]. The causes of anaemia in children are multifactorial and include malaria. In regions that are highly malaria endemic, malaria is one of the most common causes of childhood anaemia; however, severe anaemia can augment malaria morbidity and mortality in these regions [3]. Young children are yet to develop an immunity to malaria, therefore are more vulnerable. This is observed in the 2018 total malaria deaths worldwide, of which 67% were young children [4]. A significant proportion of these deaths are likely due to anaemia, directly or indirectly [5].

Even though significant progress in the fight against malaria has been made over the past two decades, more recent years has seen a levelling off to the progress, where some high-burden countries in Africa have seen a surge in the number of malaria cases and deaths [4]. Kenya, Malawi, Tanzania and Uganda were among the 19 countries that contributed to nearly 85% of the total malaria cases globally in 2018 [4]. Tanzania and Uganda saw an increase in the number of malaria cases between 2016 and 2017 and were consequently included in the High Burden to High Impact (HBHI) initiative which was launched in 2018 by the World Health Organization (WHO) and the Roll Back Malaria (RBM) Partnership to End Malaria [4]. The HBHI is a country-led approach to bring the 11 highest malaria burden countries back on track to achieving the goals of the Global Technical Strategy for Malaria 2016-2030 (GTS) of reducing malaria cases and deaths by at least 40% by 2020, at least 75% by 2025 and at least 90% by 2030 [6]. As a result, Uganda saw a significant decrease in the number of malaria cases in 2018; however, both Uganda and Tanzania still have a long way to go before reaching the GTS goals [4].

Anaemia in young children has previously been recommended as a key indicator to monitor the burden of malaria and the progress of malaria control; however, recent years has seen a decline in the awareness and reporting of this indicator [7]. The surveillance of anaemia poses challenges due to its multiple causes in children [8]. In addition, the relationship between malaria and anaemia can be confounded by several factors, including nutritional deficiencies (specifically iron deficiency) and intestinal parasites, all of which contribute to anaemia in children [5]. Although the global burden of anaemia has improved significantly since 1990, anaemia in children has shown much less improvement, thus revealing inconsistencies in the efforts to prevent childhood anaemia [9]. This may also be attributed to the complex multifactorial causes of anaemia in children which require a solid understanding of their contribution to childhood anaemia. More specifically, an understanding of the underlying causes and their relationship with anaemia in high-burden regions will aid in formulating a more targeted approach for anaemia control.

Many studies have considered the determinants of anaemia and malaria in children separately [1, 10, 11], and others have considered them as determinants of each other where children who tested positive for malaria were more than 3 times as likely to have anaemia. On the other hand, researchers have reported that those with anaemia were more than twice as likely to have malaria [12–16]. This demonstrates the association between the two outcomes; however, modelling the two jointly would reveal more about their relationship.

In this study, we made use of a joint model approach to explore the correlation between anaemia and malaria in young children across the districts or counties of four contiguous sub-Saharan African countries, namely Kenya, Malawi, Tanzania and Uganda, while accounting for the effects of socio-economic, demographic and environmental factors. In addition, we made use of maps to visualise the relationship between the two responses across the districts of the countries. To our knowledge, no studies have jointly modelled anaemia and malaria in children in these four countries. Thus, this study contributes to a better understanding of the relationship between anaemia and malaria in children in these regions of sub-Saharan Africa.

## Methods

### Study area and data

We used the data collected in the Demographic and Health Surveys (DHS) and/or the Malaria Indicator Surveys (MIS) from each of the four countries. Specifically, the data from the 2015 Kenya Malaria Indicator Survey, the 2017 Malawi Malaria Indicator Survey, the 2015-2016 Tanzania Demographic and Health Survey and Malaria Indicator Survey and the 2016 Uganda Demographic and Health Survey. These nationally represented surveys were designed to collect information on key indicators for monitoring and impact evaluation in the areas of population, health and nutrition by means of multiple questionnaires such as a household questionnaire, woman’s questionnaire and man’s questionnaire. In addition, with the consent of a parent or guardian in the sampled households, all children between the ages of 6 and 59 months were tested for anaemia and malaria using blood specimens collected from a finger- or heel-prick.

### Study variables

The two outcomes of interest were the child’s anaemia status and malaria status, where both responses were binary. The child’s anaemia status was based on the WHO definition for anaemia in children aged 6 to 59 months, where they were considered anaemic if their haemoglobin concentration, as measured using a portable HemoCue analyser, was under 11 g/dl after adjusting for altitude [17]. The child’s malaria status was based on their rapid diagnostic test (RDT) result. This consisted of testing a drop of blood using the SD Bioline Pf/Pv RDT, which tests for the presence of the *Plasmodium* parasite. This type of test has become more widely used as a diagnostic test where a reliable microscopy test is not available [18].

**Fig. 1:**
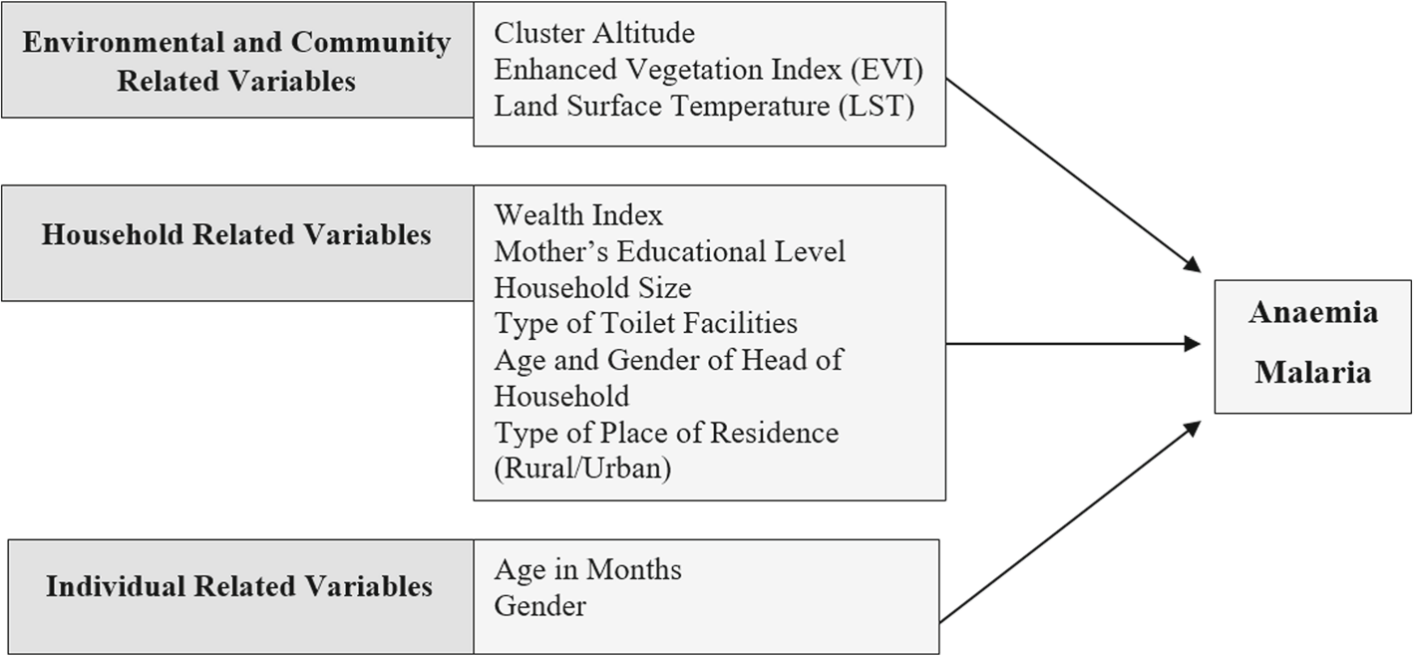
Potential risk factors of anaemia and malaria among young children

The explanatory variables considered in this study were based on those found in literature to have some association with anaemia and/or malaria, as well as those expected to be determinants of each outcome. These variables, which are displayed in Fig. 1, comprised of a number of demographic, socio-economic and environmental factors, including the gender and age of the child, the mother’s highest education level, the number of members in the household (size of the household), the type of place of residence (rural or urban), the household wealth index, the type of toilet facility, the age and gender of the head of the household and the three environmental factors: cluster altitude, day land surface temperature and the enhanced vegetation index, as well as the country of residence. The household wealth index was based on the composite measure of a household’s cumulative living standard and was calculated according to the ownership of various household assets [19]. The household was assigned a standardised score for each asset, then the scores were summed for each household to obtain a household wealth index *Z*-score, which is a continuous measure and the form of the wealth index used in this study. The two environmental factors, average day land surface temperature (LST) and the average enhanced vegetation index (EVI) for 2015, were considered as they serve as proxies for intestinal parasites, which is a risk factor for childhood anaemia [20]. Moreover, these environmental factors also impact malaria transmission as they affect both the *Plasmodium* parasite and the host (the *Anopheles* mosquito). *Plasmodium* parasites are sensitive to changes in temperature where their development slows with a drop in temperature and stops at high temperatures [21]. However, rainfall expands the breeding ground of the mosquito and also indirectly contributes to the longevity of the adult mosquito by increasing relative humidity [22]. In this study, we used the enhanced vegetation index as an indicator for rainfall, as it is correlated with rainfall [23].

### Statistical method

We propose the use of a bivariate copula regression model to jointly model anaemia and malaria. The model is based on a pair of responses and a copula specification for the dependence structure between the two responses [24]. Copulas are functions that enable the separation of the marginal distributions from the dependence structure of a given multivariate distribution [25]. The application of copula regression is diverse. McNeil et al. [26] demonstrated its use in quantitative risk management, Smith et al. [27], Madson and Fang [28], and Kürüm et al. [29] extended the application of copula regression to longitudinal data, where the approach used by Kürüm et al. [29] allowed for the model parameters to vary with time. De Leon and Chough [30] discuss further applications of copula regression to jointly model discrete as well as mixed outcomes. In addition, copula regression is commonly used in finance and insurance ([25, 31, 32], and references therein).

#### Bivariate copula regression

Suppose *Y*_*i*1_ is the anaemia status of the *i*th child and *Y*_*i*2_ is the malaria status of the *i*th child. In this study, each response is binary where *Y*_*ij*_=1 if the child had anaemia or malaria; otherwise, *Y*_*ij*_=0,*j*=1,2. The joint probability of event (*Y*_*i*1_=1,*Y*_*i*2_=1), conditional on a set of covariates ***x***_*i*1_ and ***x***_*i*2_, is defined as follows:

1 $$ \begin{aligned} P\left(Y_{i1}=1, Y_{i2}=1|\boldsymbol{x}_{i1},\boldsymbol{x}_{i2}\right) =C\left(P\left(Y_{i1}=1|\boldsymbol{x}_{i1}\right),P(Y_{i2}=1|\boldsymbol{x}_{i2});\theta\right). \end{aligned}  $$

*C* : [0,1]^2^→[0,1] is a two-place copula function and *θ*, known as the copula parameter, is an association parameter which measures the dependence between the two random variables [33]. If *Y*_*i*1_ and *Y*_*i*2_ were both continuous, the copula *C* would be unique. However, in the case of both outcomes being binary, the copula is no longer uniquely defined [24]. As such, we make use of the latent (unobserved) variable representation of binary models where we define a continuous latent variable $Y^{\ast }_{ij}=\eta _{ij}+\varepsilon _{ij}$, where *η*_*ij*_ is the linear predictor consisting of fixed and random effects as well as non-linear and spatial effects, and *ε*_*ij*_ is an error term. Therefore, *Y*_*ij*_ can be regarded as an indicator variable such that

2 $$\begin{array}{*{20}l} P\left(Y_{ij}=1|\boldsymbol{x}_{ij}\right) &=P\left(Y^{\ast}_{ij}>0|\boldsymbol{x}_{ij}\right)  \\ &=P\left(\eta_{ij}+\varepsilon_{ij}>0|\boldsymbol{x}_{ij}\right)  \\ &=P\left(\varepsilon_{ij}>-\eta_{ij}|\boldsymbol{x}_{ij}\right)  \\ &=1-F_{j}\left(-\eta_{ij}\right),  \end{array} $$

where *F*_*j*_(·) is the cumulative distribution function (CDF) of a standardised univariate distribution [33]. The copula approach allows for the specification of different families for each marginal distribution. In this study, we used the standard normal distribution for the marginal distribution of each latent response variable $Y^{*}_{ij}$, leading to a probit model. Although using a logit link would not lead to different conclusions, we selected the probit specification as it is computationally less demanding. Equation 2 can be represented as

3 $$\begin{array}{*{20}l} P\left(Y_{ij}=1|\boldsymbol{x}_{ij}\right) & = \Phi\left(\eta_{ij}\right), \end{array} $$

where *Φ*(·) is the CDF of a standard normal distribution. Therefore, a unit increase in the covariate *x*_*ijk*_ leads to a *β*_*jk*_ increase in the *Z*-score for the probability of $Y^{*}_{ij}=1$. Thus, higher values of the estimated coefficients mean that the event is more likely to happen.

#### Marginal model specification

In this study, for each marginal model, we considered the non-linear effects of the continuous covariates. We incorporated an independently and identically distributed random effect based on the district in which the child resided. This random effect, also referred to as an unstructured spatial effect, accounts for the correlation in the observations due to unmeasured district-specific factors. In other words, it accounts for the possibility that children residing in the same district would be more alike than those from different districts. In addition, we further accounted for spatial variation and spatial autocorrelation in the observations by incorporating a structured spatial effect, which accounts for the assumption that children residing in neighbouring districts are more likely to have correlated observations. We also incorporated fixed effects of all the categorical variables as well as the continuous covariates that did not display a strong non-linear effect on each response. The resulting model for each response takes the form of a geoadditive mixed model, which is an extension of a generalised additive mixed model (GAMM) [34]. Each marginal model can consist of different effects. The non-linear effects were estimated by smooth functions using a regression spline approach, and the structured spatial effect was estimated using a Markov random field smoother, which was based on the neighbourhood structure of the districts across the four countries. Two districts are considered neighbours if they share a border. More information on the specification and estimation of each marginal model can be found in [24].

#### Copula specification

An advantage of the copula approach to joint modelling is that the selection of the copula for modelling the dependence between the outcomes is independent of the choice of the marginal distributions [35]. Several different types of copulas exist, of which the most common are discussed in [36] and [37]. To choose the most appropriate copula, information criteria such as the Akaike information criterion (AIC) and Bayesian information criterion (BIC) are used, where the copula producing the lowest of these values is selected. In our study, the Frank copula produced the smallest AIC value and thus was selected to jointly model our responses. The Frank copula is of the Archimedean class and has the following form:

4 $$ \begin{aligned} C\left(F_{1}\left(Y_{i1}\right),F_{2}\left(Y_{i2}\right);\theta\right) = -\frac{1}{\theta} \ln \left[1+\frac{\left(e^{-\theta \times F_{1}}-1\right)\left(e^{-\theta \times F_{2}}-1\right)}{e^{-\theta}-1}\right]. \end{aligned}  $$

The copula parameter, *θ*, is not straightforward to interpret. Therefore, it can be converted into the Kendall correlation coefficient, or Kendall’s tau (*τ*∈[−1,1]), which is a measure of the degree of concordance [33]. For the Frank copula, *τ* can be obtained by solving the following equation:

5 $$ \frac{D_{1}(\theta)-1}{\theta}=\frac{1-\tau}{4},  $$

where

6 $$ D_{1}(\theta)=\frac{1}{\theta}\int_{0}^{\theta}\frac{t}{e^{t}-1}dt.  $$

If *τ*=0, then *Y*_*i*1_ and *Y*_*i*2_ are independent. The Frank copula is comprehensive, which means it covers the full spectrum of possible values of *τ*, which is not the case for all copulas [38].

The copula parameter, *θ*, may also vary according to different groups of observations. Therefore, *θ* can be specified as a function of a linear predictor, such as *θ*_*i*_=*m*(*η*_*i*3_), where *m* is a one-to-one transformation that ensures that *θ*_*i*_ lies in its range, and *η*_*i*3_ is the linear predictor associated with the copula parameter [33]. The transformation applied depends on the specified copula function. This framework allows one to explore the association between the two outcomes according to the levels or categories of certain factors. In this study, we varied the copula parameter according to the district of residence to enable us to determine the districts in which there is a strong association between anaemia and malaria. Conversely, we are also able to determine the districts in which the association is weak, therefore suggesting that there are other significant drivers of anaemia in children in those districts.

We used the R package *GJRM* (Generalised Joint Regression Modelling) for the analysis [39]. The mapping of the results was done in QGIS 3.4 (https://qgis.org/en/site/index.html), and all the maps created were based on our results by making use of shapefiles freely available from the DHS Program’s Spatial Data Repository (https://spatialdata.dhsprogram.com/boundaries).

## Results

### Sample characteristics

The total sample size combined was 18196 children from the four countries. Table 1 shows the observed anaemia and malaria prevalence. The observed prevalence of anaemia from the four countries was 52.5%, while the malaria prevalence was 19.7%, with a 15.1% prevalence of both anaemia and malaria. The uncorrected Kendall’s tau correlation between anaemia and malaria was estimated at 0.239, which was statistically significant at a 5% significance level.

**Table 1:** Cross-tabulation of the sample according to anaemia and malaria status

		Result of malaria rapid test		Total
		Positive	Negative	
Anaemia status	Anaemic	2750 (15.1)	6809 (37.4)	9559 (52.5)
	Non-anaemic	842 (4.6)	7795 (42.8)	8637 (47.5)
	Total	3592 (19.7)	14604 (80.3)	18196

Table 2 presents the observed prevalence of anaemia, malaria and both anaemia and malaria according to the categorical variables of interest. To aid in the assessment of anaemia as a public health problem, anaemia was categorized into four by the WHO, where it is considered a severe health problem if the prevalence is 40% or more, moderate from 20 to 39.9%, mild from 5 to 19.9%, and no public health problem if the prevalence is less than or equal to 4.9% [40]. According to these classifications, Malawi, Tanzania and Uganda have a severe public health problem. Kenya had the lowest observed prevalence of anaemia (38.3%), malaria (9.3%) and both (6%) in children. No large differences in the prevalence of anaemia or malaria or both were seen between male and female children, as well as between children in households headed by males or females. The observed prevalence of anaemia, malaria and both decreased with an increase in education level as well as with an improvement in the type of toilet facility. A considerably higher observed prevalence of malaria as well as both anaemia and malaria was seen in children residing in rural areas compared to those in urban areas.

**Table 2:** The distribution of children by outcome according to the categorical explanatory variables

Variable	Sample size	Anaemia (%)	Malaria (%)	Both (%)
*Country*				
Kenya	3424	1311 (38.3)	317 (9.3)	206 (6.0)
Malawi	2270	1323 (58.3)	601 (26.5)	459 (20.2)
Tanzania	7819	4408 (56.4)	1099 (14.1)	900 (11.5)
Uganda	4683	2517 (53.7)	1575 (33.6)	1185 (25.3)
*Gender*				
Male	9143	4927 (53.9)	1821 (19.9)	1410 (15.4)
Female	9053	4632 (51.2)	1771 (19.6)	1340 (14.8)
*Type of place of residence*				
Urban	4605	2160 (46.9)	292 (6.3)	220 (4.8)
Rural	13591	7399 (54.4)	3300 (24.3)	2530 (18.6)
*Mother’s highest education level*				
No education	2893	1744 (60.3)	705 (24.4)	561 (19.4)
Primary	9757	5253 (53.8)	2013 (20.6)	1565 (16.0)
Secondary and higher	3110	1444 (46.4)	290 (9.3)	194 (6.2)
Unknown	2436	1118 (45.9)	584 (24.0)	430 (17.7)
*Type of toilet facilities*				
No toilet facility	2367	1462 (61.8)	641 (27.1)	521 (22.0)
Pit latrine	14587	7564 (51.9)	2914 (20.0)	2202 (15.1)
Flush toilet	1242	533 (42.9)	37 (3.0)	27 (2.2)
*Gender of head of household*				
Male	13869	7342 (52.9)	2736 (19.7)	2119 (15.3)
Female	4327	2217 (51.2)	856 (19.8)	631 (14.6)

**Fig. 2:**
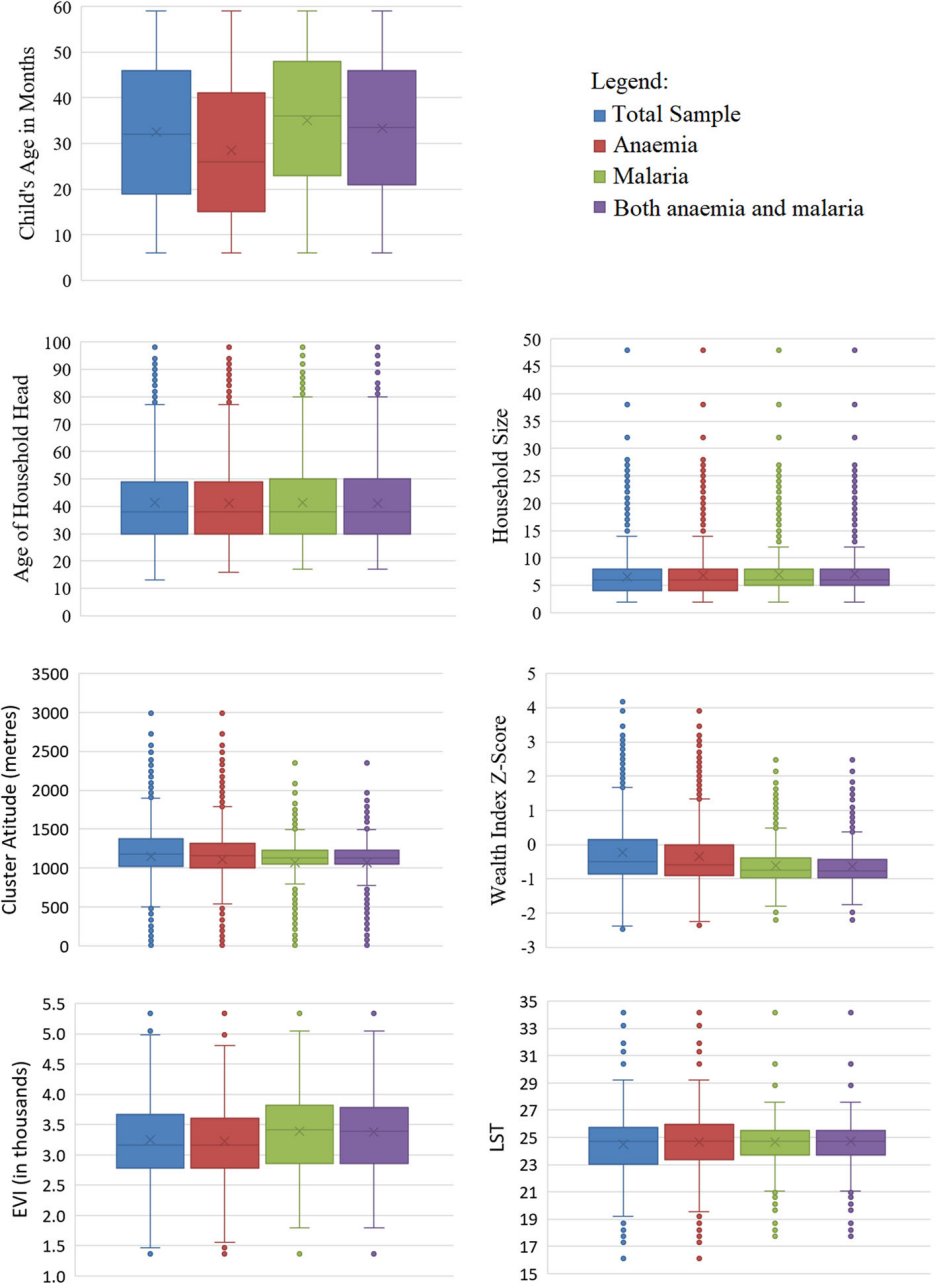
Boxplots for the continuous covariates by the outcome categories

Boxplots for each of the continuous covariates are presented in Fig. 2. These boxplots display the minimum, first quartile, median, third quartile, maximum and the mean of each covariate based on all the children in the sample, the children with anaemia, the children with malaria and the children with both anaemia and malaria. Children with anaemia had a lower age, on average, compared to those with malaria. Not much difference in the distributions of the age of the household head and the household size was seen between the different samples of children. Children with malaria, on average, resided in clusters at a lower altitudes. On average, children with anaemia or malaria or both anaemia and malaria resided in households with a slightly lower wealth index compared to the full sample of children. The environmental factor EVI had the highest mean and median for those children with malaria. Not much difference in the mean or median of LST was evident between the samples.

### Results of the bivariate copula regression model

Prior to fitting the full bivariate copula model, univariate logistic regression was used to determine which independent variables should be selected to be entered into each marginal model for each response (anaemia and malaria) based on a relaxed *p* value of 20%, where only those with a *p* value less than 0.2 were selected. The age of the household head was the only variable not incorporated into the marginal model for anaemia, whereas the age and gender of the household head as well as the child’s gender were not incorporated into the marginal model for malaria. The non-linear effect of all continuous covariates (child’s age in months, household size, wealth index *Z*-score, cluster altitude, EVI and LST) on each response was explored. However, only the child’s age in months showed clear evidence of non-linearity on both responses; thus, it was the only non-linear effect considered. The remaining continuous covariates were incorporated into each marginal model as fixed effects.

The model did not achieve convergence with the inclusion of the country of residence as a fixed effect. We believe the effect of the country is possibly redundant with the inclusion of the spatial effects at district level, as the effect of each country can be obtained by systematic aggregation of the effects of the districts within the country. Upon removal of the country effect, the model achieved convergence and the observed information matrix was positive definite. Thus, the results presented below are based on the model excluding the effect of the country.

#### Fixed effects results

Table 3 presents the results of the fixed effects for each marginal model. Based on these results, children residing in rural areas had a lower likelihood of malaria compared to those residing in urban areas; however, there was no significant difference in the likelihood of anaemia between these children (rural estimate = − 0.020, *p* value = 0.535 for anaemia; rural estimate = 0.299, *p* value < 0.001 for malaria). The likelihood of each outcome significantly decreased with an increase in the mother’s highest education level. The type of toilet facilities was significantly associated with a child’s anaemia status, but not their malaria status, where the likelihood of anaemia decreased with an improvement of the toilet facility type (pit latrine estimate = − 0.158, *p* value < 0.001; flush toilet estimate = − 0.165, *p* value = 0.008 for anaemia). An increase in the number of household members resulted in a significantly higher likelihood of anaemia; however, it had no significant effect on a child’s malaria status (household size estimate = 0.009, *p* value = 0.006 for anaemia; household size estimate = 0.001, *p* value = 0.705 for malaria). A unit increase in the household’s wealth index *Z*-score was associated with a significant decrease in the likelihood of each anaemia and malaria (wealth index estimate = − 0.158, *p* value < 0.001 for anaemia; wealth index estimate = − 0.503, *p* value < 0.001 for malaria). Cluster altitude was significantly associated with each response, where the likelihood of each decreased with an increase in altitude (cluster altitude estimate = − 0.016, *p* value = 0.002 for anaemia; cluster altitude estimate = − 0.089, *p* value < 0.001 for malaria). EVI was significantly associated with only malaria, where an increase resulted in an increased likelihood of malaria (EVI estimate = 0.405, *p* value = 0.001 for malaria). LST was not significantly associated with either response.

**Table 3:** Parameter estimates, standard errors and *p* values of the fixed effects for the bivariate copula regression model for anaemia and malaria

Variable	Anaemia			Malaria		
	Estimate	St. error	*p* value	Estimate	St. error	*p* value
*Gender (ref = male)*						
Female	− 0.083	0.019	<0.001∗		NA	
*Type of place of residence (ref = urban)*						
Rural	− 0.020	0.032	0.535	0.299	0.047	<0.001∗
*Mother’s education level (ref = no education)*						
Primary	− 0.115	0.031	<0.001∗	− 0.125	0.039	0.001 ∗
Secondary and higher	− 0.164	0.042	<0.001∗	− 0.250	0.057	<0.001∗
Unknown	− 0.095	0.039	0.016 ∗	0.012	0.049	0.802
*Gender of household head (ref = male)*						
Female	0.011	0.024	0.633		NA	
*Type of toilet facility (ref = no facilities)*						
Pit latrine	− 0.158	0.035	<0.001∗	− 0.078	0.043	0.072
Flush toilet	− 0.165	0.062	0.008 ∗	0.102	0.114	0.366
*Household size*	0.009	0.003	0.006 ∗	0.001	0.004	0.705
*Wealth index*	− 0.158	0.019	<0.001∗	− 0.503	0.029	<0.001∗
*Cluster altitude (in 100 m)*	− 0.016	0.005	0.002 ∗	− 0.089	0.009	<0.001∗
*EVI*	0.068	0.057	0.229	0.405	0.121	0.001 ∗
*LST*	0.011	0.015	0.452	0.019	0.033	0.563

*NA* not applicable as the factor was not incorporated into the marginal model for that response

^∗^Significant at 5% level of significance

#### Non-linear and spatial effect results

**Fig. 3:**
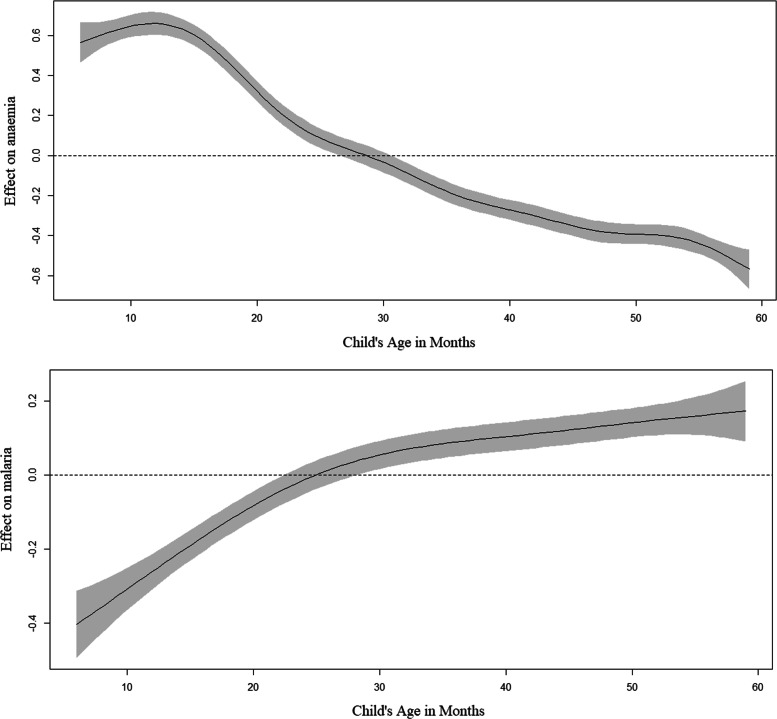
Estimated non-linear effect of the child’s age on anaemia (top) and malaria (bottom)

Table 4 displays the significance of the non-linear and spatial effects for both responses. Both the structured spatial effect and unstructured spatial effect (the district-level random effect) had a significant effect on the likelihood of each response. Further, the child’s age in months had a significant non-linear effect on the likelihood of each response. Figure 3 displays this non-linear effect that a child’s age in months had on anaemia and malaria. The likelihood of anaemia decreased with an increase in age. However, there was a reverse effect of age on malaria, where the chance of malaria increased with an increase in age.

**Table 4:** Approximate significance for the non-linear and spatial effects

Variable	Anaemia		Malaria	
	Chi-square value	*p* value	Chi-square value	*p* value
Child’s age in months	1472.50	<0.001∗	138.49	<0.001∗
Unstructured spatial effect	357.70	<0.001∗	34.75	<0.001∗
Structured spatial effect	183.80	<0.001∗	1412.17	<0.001∗

^∗^Significant at 5% level of significance

**Fig. 4:**
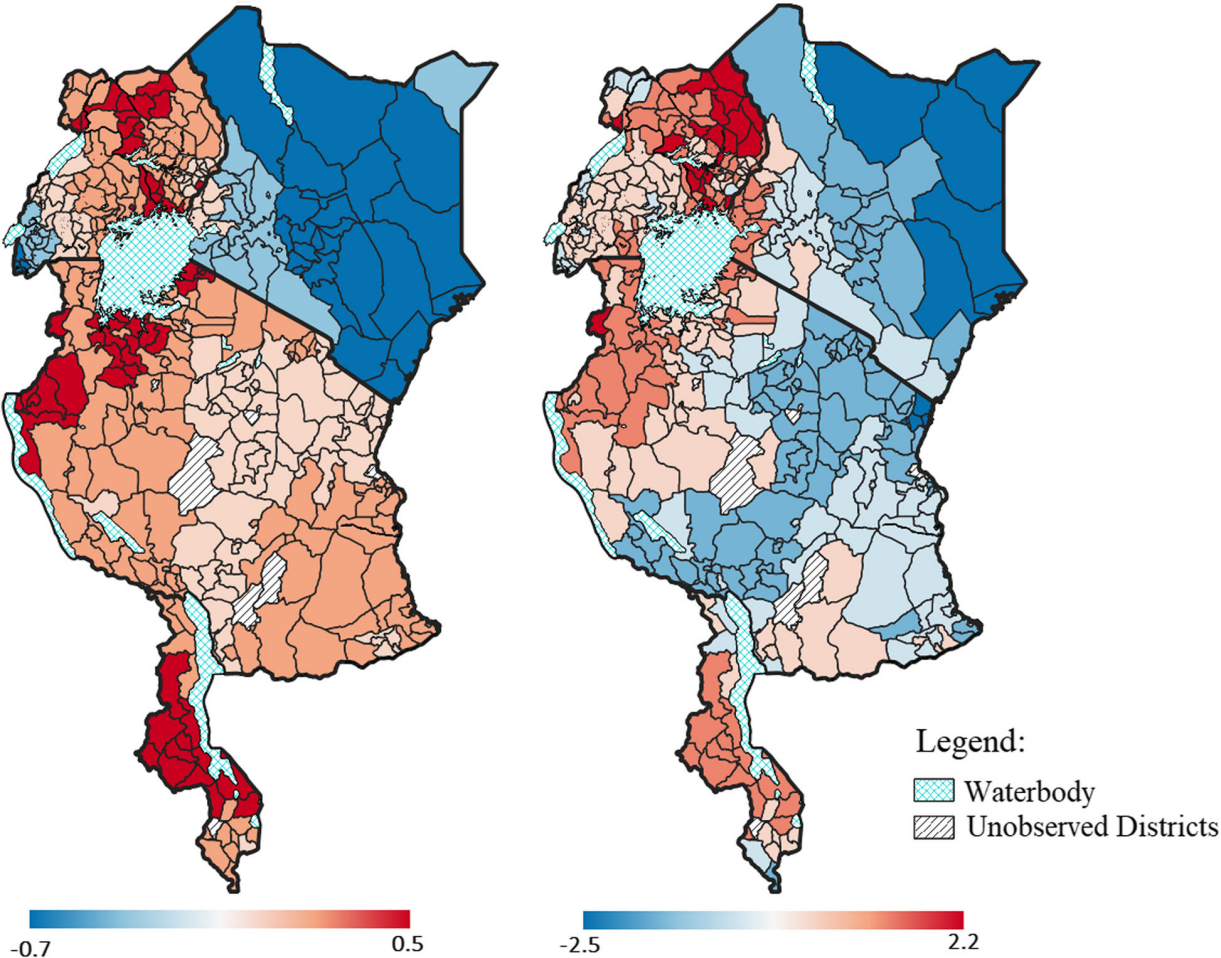
Estimated effect of the structured spatial effect on anaemia (left) and malaria (right). Top left: Uganda; top right: Kenya; middle: Tanzania; and bottom Malawi

The district-level structured spatial effect for both anaemia and malaria is presented in Fig. 4. The districts in shadings of blue correspond to a negative estimated effect and were therefore associated with a lower likelihood of the event. However, districts in shadings of red correspond to a positive estimated effect and were therefore associated with a higher likelihood of the event. There was a lot less variation observed in the structured spatial effect for anaemia compared to that for malaria. The structured spatial effect for malaria revealed that Tanzania consisted of districts associated with a lower likelihood of malaria as well as districts associated with a higher likelihood of malaria. This apparent spatial variation suggests that it was important to control for as failure to do so would reduce the statistical power of inference in the model and therefore lead to inaccurate results [41].

#### Conditional dependence of anaemia and malaria

**Fig. 5:**
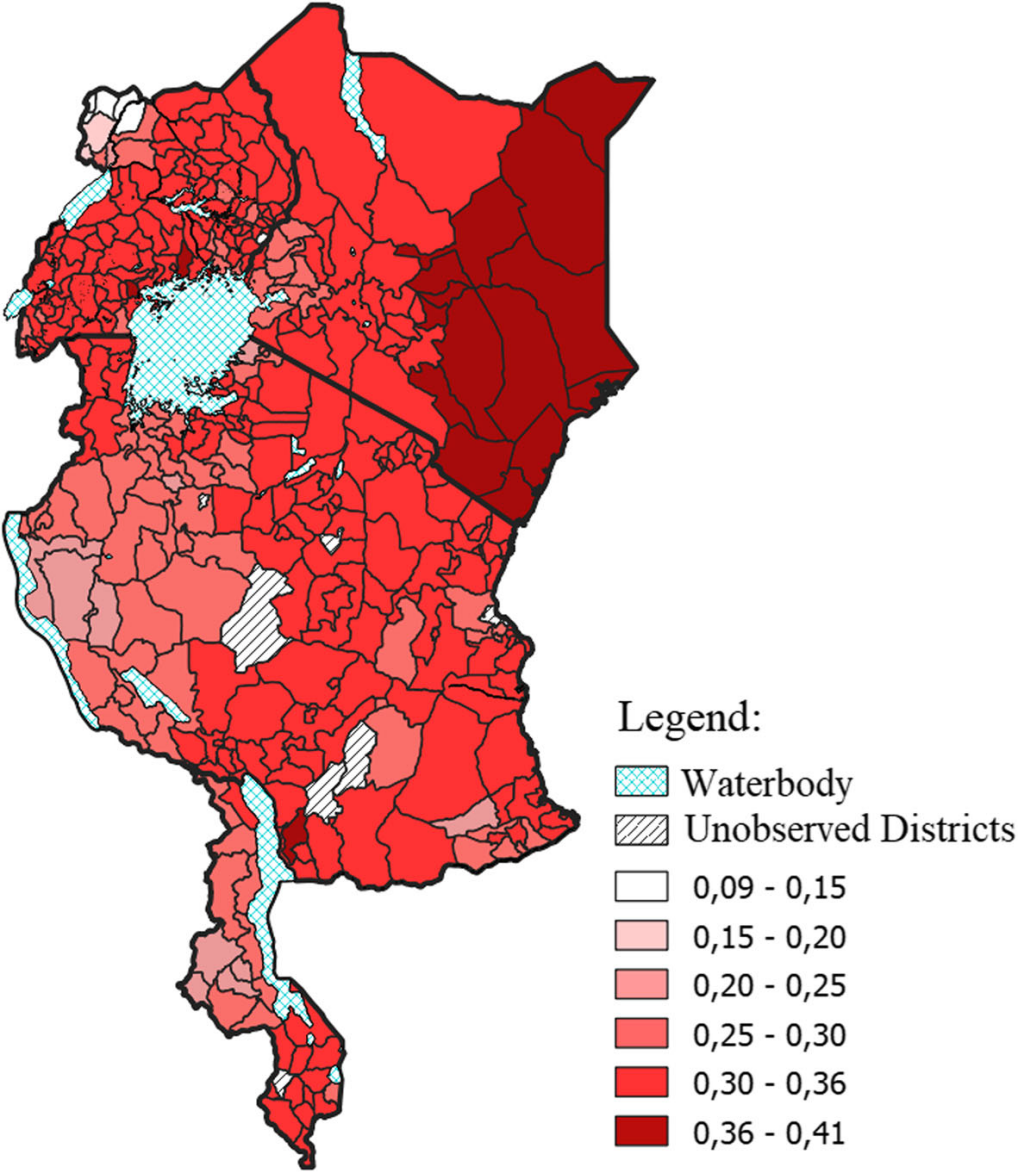
Estimated Kendall’s *τ* according to district of residence. Top left: Uganda; top right: Kenya; middle: Tanzania; and bottom Malawi

The copula parameter was set to vary according to the district/county of residence across the four countries. This was done by linking the additive predictor for the copula parameter to a Markov random field term based on these districts of residence. The estimated value of the copula parameter, averaged out over the districts, was 3.07 with a 95% confidence interval of (1.56, 4.61). This copula parameter, which was estimated conditioned on the observed covariates and spatial variation, was then used to estimate Kendall’s *τ* for each district as shown in Fig. 5. This figure displayed a fairly heterogeneous, non-zero association between anaemia and malaria in young children across the districts. With using the Frank copula, we allowed for positive and negative associations between anaemia and malaria. However, Kendall’s *τ* ranged between 0.09 and 0.41, with an average of 0.31 and a 95% confidence interval of (0.16, 0.42). Thus, there was a positive association between malaria and anaemia. A stronger association was observed in some districts compared to others. Kenya depicted more districts with the highest association.

The above result suggests that the probability of a child being anaemic or having malaria in a particular district should be based on the joint probability from the bivariate model rather than each independent univariate model. These joint probabilities can further reveal more about the relationship between anaemia and malaria in children across the districts of the four countries.

#### Estimated joint probability of anaemia and malaria

**Fig. 6:**
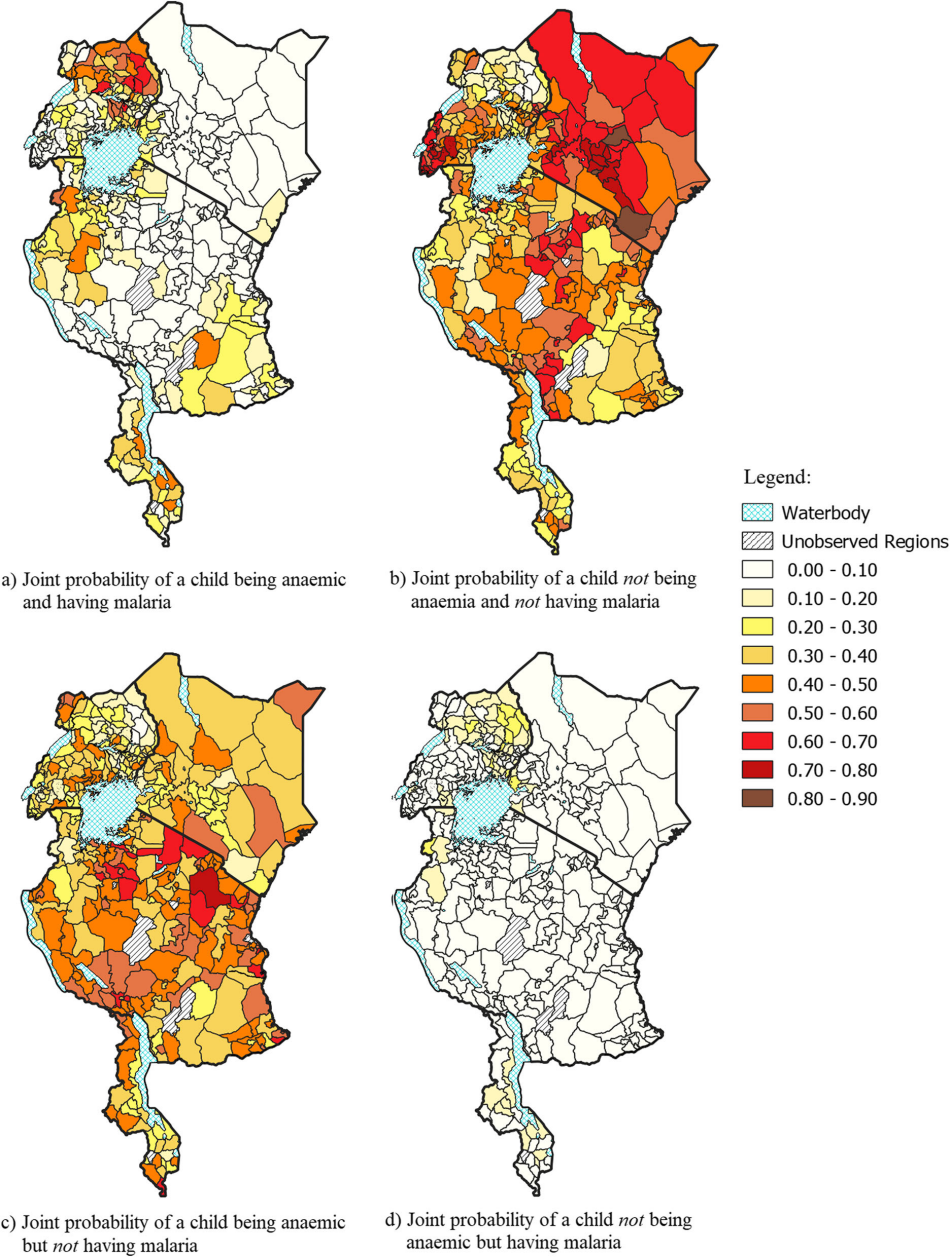
Estimated joint probabilities based on the bivariate copula regression model

Based on the fitted bivariate copula regression model, the estimated joint probabilities were extracted and averaged over the districts. Figure 6 shows these joint probabilities for each combination of outcome for anaemia and malaria in young children. On the whole, these joint probabilities were generally heterogeneous within each country.

Considering image a in Fig. 6, a large number of districts in Uganda showed a considerably high joint probability of a child having anaemia and malaria, particularly in the north/north east of the country. Kenya was homogeneous in these probabilities, which were also all fairly low (all were below 0.20). Malawi had a few districts with a relatively high probability of both anaemia and malaria in children. From image b, we can observe that the majority of districts in Kenya had a high probability of a child not having anaemia nor malaria. This is unsurprising as Kenya also had the lowest observed prevalence of anaemia and malaria.

Paying particular attention to image c, throughout the districts considered in each country, there were a fair number that displayed a high chance of a child having anaemia but not malaria. In these districts, it would be inaccurate to use anaemia as an indicator for malaria as this image suggests that there are other significant drivers of anaemia in children in these districts. Image d reveals very low probabilities of a child having malaria but not anaemia throughout the majority of the districts. In other words, it is highly unlikely for a child to have malaria but not anaemia in these districts. Thus, it is clear that there is a high likelihood of a child developing anaemia when they have malaria. Based on images a and d, districts in the northern part of Uganda had a relatively high probability of a child having malaria, regardless of anaemia status. This is also supported by Uganda having the highest observed prevalence of malaria.

## Discussion

This study aimed to explore the relationship between anaemia and malaria in young children across the districts/counties of Kenya, Malawi, Tanzania and Uganda by making use of a joint bivariate copula regression model. This approach allows the correlation between the two responses to be estimated while controlling for the linear and non-linear effects of independent variables, as well as the effect of spatial variation. The copula framework allows the dependency structure between the responses to be isolated from their marginal distributions. The advantage of copula regression over multivariate analysis is that normality and linearity of the dependence between the responses is not assumed. In fact, in general, dependence in copulas is non-linear [38]. Further, the appeal of the copula approach is that one is able to vary the association between the responses according to the different levels of certain factors, rather than obtaining one estimated value for the correlation as is the case with a joint multivariate model [42].

We varied the association according to the district of residence. This revealed a positive association between anaemia and malaria throughout the districts, however the strength of which varied across the districts of the four countries. Some districts had a stronger association between the two responses compared to other districts. While we are interested in the likelihood of a child having both anaemia and malaria, considering the likelihood of all combinations of outcomes of these events can further aid in better understanding the relationship between anaemia and malaria. Therefore, we made use of the estimated joint probabilities for the combination of outcomes, which we mapped across the districts. These maps generally indicated a variation in the joint probabilities within each country. This suggests that any approach to anaemia or malaria control should be targeted rather than a country-wide approach. Districts in the north to north east part of Uganda displayed high probabilities of a child having malaria, for both those with or without anaemia. These districts need an up-scaled targeted approach to malaria control. Districts in Kenya showed the least amount of variation in some of the joint probabilities and also had the lowest joint probability associated with a child having malaria, for those with or without anaemia. This is as a consequence of the major progress that Kenya has made in the fight against malaria, which is most likely owed to the recent malaria prevention measures that have been tailored to local needs [43].

If anaemia is to be used as an indicator for the success of malaria control programmes, in any country, it would only be useful in areas where there is a strong correlation between anaemia and malaria as well as a high probability of the two. Thus, maps created in this study aid in identifying such areas. In addition, based on the map of the joint probability of a child having anaemia but not malaria, a high likelihood of this event was revealed in many of the districts. In such districts, it would be reasonable to assume that there are other drivers of anaemia in children, other than malaria. Therefore, applying malaria interventions in these districts to aid in the reduction of the prevalence of childhood anaemia would be ineffective. Further investigation into the drivers of childhood anaemia in these districts is therefore required.

The results of the effects considered in this study are consistent with those from other studies that modelled anaemia and malaria separately, where the child’s age, mother’s education level, household wealth index and cluster altitude were significantly associated with both anaemia and malaria status [10, 11, 44, 45]. The child’s gender, the household size and the type of toilet facility were further significantly associated with anaemia in children, as seen in other studies [46, 47]. No toilet facility or unimproved toilet facilities (such as an open pit or bucket) can lead to poor sanitation, which creates an environment supportive of hookworms, an intestinal parasite that contributes to anaemia in children [48].

Very few studies have jointly modelled anaemia and malaria. The studies that have done so have also utilised different techniques and thus answered different questions [3, 49]. A bivariate probit model was used to jointly model anaemia and malaria in individuals between the ages of 15 and 60 in Alaba District, Southern Ethiopia, the result of which showed a positive correlation between malaria and anaemia; however, the magnitude of the correlation was not explored [49]. Similar to our study, [3] jointly modelled anaemia and malaria in children under 5 in Nigeria and found substantial geographical variations in the likelihood of malaria; however, the association between anaemia and malaria was not directly explored.

As multiple factors were significantly associated with both anaemia and malaria, accordingly, we propose further varying the association parameter by the levels of these factors. For example, the additive predictor for the copula parameter can include the effects of the mother’s education level in addition to the district-level structured spatial effect. The correlation and joint probabilities can then be estimated according to the levels of the additional factors, which will further reveal more about the relationship between anaemia and malaria.

A limitation of this study includes the use of cross-sectional data; thus, a causal relationship could not be determined. Furthermore, a lack of data on important factors of anaemia in children, such as iron deficiency and intestinal parasites, may restrict the findings of this study.

## Conclusion

This study presents an alternative technique to joint modelling of anaemia and malaria in young children which assists in understanding more about their relationship compared to techniques of multivariate modelling. The approach used in this study can aid in visualising the relationship through mapping of their correlation and joint probabilities. These maps produced can then help policy makers target the correct set of interventions, or prevent the use of incorrect interventions, particularly for childhood anaemia, the causes of which are multiple and complex.

## Data Availability

This study utilised existing survey datasets that are in the public domain and freely available from http://www.dhsprogram.com/data/dataset_admin/login_main.cfmwith the permission from the DHS Program.

## Abbreviations

95% CI: 95% confidence intervals
AIC: Akaike information criterion
BIC: Bayesian information criterion
CDF: Cumulative distribution function
DHS: Demographic and Health Survey
EVI: Enhanced vegetation index
GAMM: Generalised additive mixed model
GTS: Global Technical Strategy for Malaria 2016-2030
HBHI: High Burden to High Impact
LST: Land surface temperature
MIS: Malaria Indicator Survey
RDT: Rapid diagnostic test
WHO: World Health Organization
